# Real-Time ECG Artifact Removal for Adaptive Deep Brain Stimulation: A Comparative Study

**DOI:** 10.3390/s26092673

**Published:** 2026-04-25

**Authors:** Lucrezia Silvi, Valentina D’Onofrio, Simone Cauzzo, Angelo Antonini, Andrea Guerra, Camillo Porcaro

**Affiliations:** 1 Biomedical Engineering Research to Advance and Innovate Translational Neuroscience (BRAIN Unit), Department of Neuroscience, University of Padova, 35122 Padua, Italy; lucrezia.silvi@studenti.unipd.it (L.S.); simone.cauzzo@unipd.it (S.C.); 2 Padova Neuroscience Center (PNC), University of Padova, 35122 Padua, Italy; valentina.donofrio@studenti.unipd.it (V.D.); angelo.antonini@unipd.it (A.A.); andrea.guerra@unipd.it (A.G.); 3 Department of Neuroscience, University of Padova, 35122 Padua, Italy; 4 Istituto di Ricovero e Cura a Carattere Scientifico (IRCCS), San Camillo Hospital, 30126 Venice, Italy; 5 Institute of Cognitive Sciences and Technologies (ISTC), National Research Council (CNR), 00185 Rome, Italy; 6 Center of Human Brain Health, School of Psychology, University of Birmingham, Birmingham B15 2TT, UK

**Keywords:** adaptive deep brain stimulation, ECG artifact, real-time signal processing, local field potentials, Parkinson’s disease

## Abstract

**Highlights:**

**What are the main findings?**

- ECG artifact removal using template subtraction combines high biomarker fidelity (beta band preservation) with strict real-time feasibility on sensing-enabled DBS systems.
- More aggressive methods (e.g., eSVD, Perceive) achieve stronger artifact suppression but fail real-time computational constraints and degrade biomarker reliability under realistic conditions.

**What are the implications of the main findings?**

- Real-time adaptive DBS systems should prioritize balanced artifact removal strategies that preserve beta power stability rather than maximizing artifact suppression alone.
- Consequently, Template Subtraction emerges as the most clinically and computationally viable solution for direct integration into the low-power embedded firmware of next-generation closed-loop neurostimulators.

**Abstract:**

Background: Adaptive deep brain stimulation (aDBS) for Parkinson’s disease (PD) relies on accurate detection of beta oscillatory activity. However, electrocardiographic (ECG) artifacts frequently contaminate local field potentials (LFPs), compromising control algorithms. While offline cleaning methods exist, their feasibility for real-time operation within the strict timing constraints of current sensing-enabled devices remains unknown. Methods: We evaluated four ECG removal algorithms, template subtraction (TS), singular value decomposition (SVD), extended SVD (eSVD), and the Perceive toolbox (PR), on simulated datasets (contaminated at −30 to +20 dB) and clinical recordings from 20 PD patients. Algorithms were assessed for artifact removal quality (beta power preservation, signal-to-noise ratio) and real-time feasibility (99th percentile processing latency—P_99_ < 50 ms). Results: Only TS and standard SVD met the real-time feasibility threshold, with TS achieving superior timing consistency (P_99_ ≈ 10 ms). eSVD and PR proved incompatible with closed-loop requirements (P_99_ > 90 ms). While eSVD yielded the highest artifact suppression at extreme contamination, it suffered from poor signal preservation at moderate levels. TS demonstrated the best balance, maintaining beta power accuracy within ±12% across clinically relevant contamination levels. Conclusions: TS is the recommended method for real-time aDBS applications, offering a safety-critical balance of computational efficiency and biomarker fidelity.

## 1. Introduction

Deep brain stimulation (DBS) is an effective treatment for patients with advanced Parkinson’s disease (PD), significantly improving motor function and quality of life [1,2]. High-frequency stimulation (100–180 Hz) suppresses local pathological beta oscillations (13–30 Hz) in the target structures, with beta power reduction strongly correlating with improvements in cardinal motor symptoms [3,4]. In PD, beta activity also exhibits a distinctive temporal structure [5,6], as pathological beta oscillations occur in phasic bursts, and treatment-related clinical improvements are paralleled by a decrease in burst duration and amplitude [6,7,8].

Conventional DBS (cDBS) operates in an open-loop manner, delivering continuous stimulation irrespective of moment-to-moment symptom fluctuations [9,10]. Although effective, this approach presents some limitations: it may lead to overstimulation, fails to respond to changes in symptoms during the day, and drains the device’s battery faster, requiring more frequent replacements of implantable pulse generators (IPGs) [7,10,11,12]. To overcome these limitations, closed-loop adaptive DBS (aDBS) systems that dynamically adjust stimulation based on real-time neural biomarkers have been recently introduced in clinical practice for PD [9,10]. The available aDBS systems allow simultaneous stimulation delivery and local field potential (LFP) sensing from brain targets [5,13,14]. Beta oscillations currently represent the control signals for aDBS systems, considering their established role as symptom biomarkers. Despite these encouraging clinical results, the reliability of the neural biomarker and its accurate tracking throughout the day remain critical challenges for successful aDBS implementation [15,16,17].

However, the robust implementation of beta-based aDBS is frequently compromised by electrocardiographic (ECG) artifacts [18,19,20]. LFP recordings used to extract the beta band biomarker are typically referenced to the thoracic IPG case [18,21]. This configuration creates a relatively large recording dipole spanning the thorax, and recorded LFPs are particularly susceptible to cardiac far-field activity, resulting in pronounced ECG contamination [18,19]. Furthermore, the duration of the QRS complex (150–200 ms), its frequency (60–100 bpm), and its spectral energy largely overlap with the beta band, posing significant challenges for biomarker estimation [18,19,20]. Indeed, previous works [19] showed that ECG-contaminated LFP recordings exhibited a dramatic increase in beta power compared to non-contaminated signals, rendering raw recordings unsuitable for aDBS control. While several ECG suppression techniques have shown promise in offline post-processing [19,20,22] a critical gap remains concerning their suitability for real-time application. The temporal structure of beta activity is also a critical factor for aDBS control algorithms. Since pathological beta bursts last 200–1000 ms, effective feedback control systems must update much faster than this window [7,23]. For instance, control windows of 400 ms allow for the selective targeting of pathological bursts while preserving physiological activity [10,23]. Current sensing-enabled neurostimulators update biomarker estimates every 100 ms [5,13,14], requiring artifact removal algorithms to complete processing within stringent latency budgets. To date, no study has systematically evaluated whether existing ECG suppression methods can meet these real-time computational requirements.

While prior literature has established the offline efficacy of various ECG removal algorithms, a critical translational gap remains: unconstrained offline performance does not equate to real-time closed-loop viability [19]. The primary novelty of this study is the introduction of a buffer-by-buffer computational benchmarking framework to evaluate these algorithms against the strict 100 ms temporal constraints of embedded aDBS systems. Furthermore, by utilizing a medication-OFF baseline, our simulation framework provides a highly robust test of beta biomarker preservation compared to previous medication-ON simulations. The significance of this work lies in challenging the offline assumption that SVD-based artifact suppression is universally superior, demonstrating instead that, for real-time neuromodulation, algorithmic simplicity (template subtraction) and biomarker preservation must supersede exhaustive artifact elimination.

## 2. Materials and Methods

### 2.1. Study Population and Data Acquisition

Twenty patients with PD (7 females, mean age ± standard deviation 62.7 ± 6.2) treated with bilateral DBS targeting either the subthalamic nucleus (STN, *n* = 12) or globus pallidus internus (GPi, *n* = 8) were included. All patients were implanted with the sensing-enabled Medtronic Percept™ PC neurostimulator and quadripolar segmented leads (model B33005) (Medtronic Inc., Minneapolis, MN, USA) [5,13]. A total of 36 hemispheres from 18 patients were considered for analysis; two patients were excluded due to movement-induced artifacts rather than ECG contamination, ensuring that the most difficult cardiac cleaning scenarios were retained in the analysis. Each hemisphere was analyzed independently. Of the 18 patients included, 3 were used for simulated data analysis, yielding 33 test signals (3 patients × 11 contamination levels), and all 18 for real data validation. All participants provided written informed consent prior to enrollment. Study procedures were conducted in accordance with the Declaration of Helsinki.

LFP recordings were obtained across four experimental conditions resulting from the combination of different medication and stimulation states. All patients were evaluated after overnight L-Dopa withdrawal (>12 h, M0) and 1 h after L-Dopa administration (M1). Stimulation state comprised S0 (DBS turned off) and S1 (DBS turned on using individual chronic stimulation parameters).

LFPs were recorded for ≈120 s using the BrainSense™ (Medtronic Inc., Minneapolis, MN, USA) Streaming mode from electrode contacts adjacent to the chronic stimulating level, referenced to the implanted pulse generator (IPG) case, to maximize common-mode rejection of stimulation artifacts [13]. Signals were sampled at 250 Hz and hardware-filtered (1–100 Hz) before digitization [5,19].

### 2.2. Data Analysis

**Figure 1:**
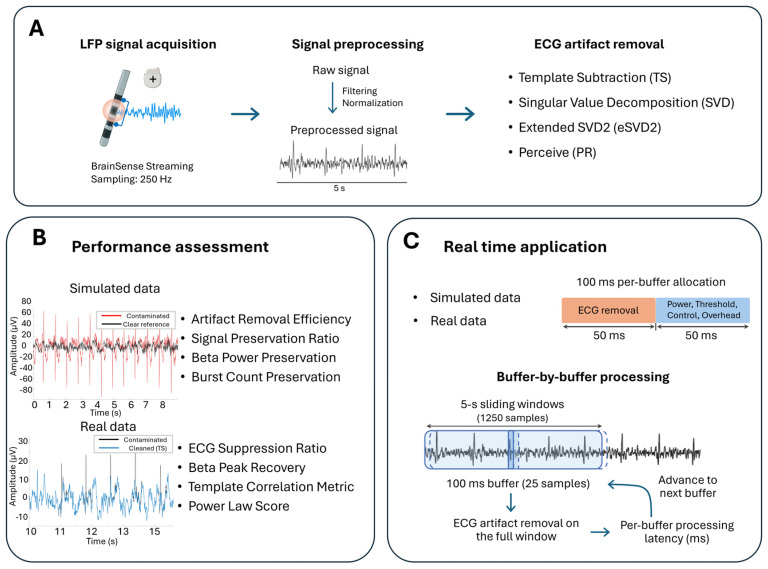
Study design overview. (**A**) LFP signal acquisition and pre-processing pipeline. Signals were acquired via BrainSense Streaming at 250 Hz, bandpass filtered and normalized, and processed using four ECG artifact removal algorithms: Template Subtraction (TS), Singular Value Decomposition (SVD), Extended SVD (eSVD), and the Perceive toolbox (PR). (**B**) Performance assessment framework. Algorithm quality was evaluated on simulated data with known ground truth using four metrics (Artifact Removal Efficiency, Signal Preservation Ratio, Beta Power Preservation, Burst Count Preservation) and validated on real clinical recordings using reference-free metrics (ECG Suppression Ratio, Beta Peak Recovery, Template Correlation Metric, Power Law Score). (**C**) Real-time feasibility framework. Buffer-by-buffer processing was implemented to simulate device-level operation: each 100 ms buffer (25 samples) was processed within a 5 s sliding context window (1250 samples), with 50 ms allocated to ECG removal and 50 ms reserved for power estimation, threshold comparison, control decisions, and system overhead.

All data were analyzed offline using MATLAB R2023b (The MathWorks Inc., Natick, MA, USA). The signal analysis pipeline comprised three main steps: (i) application of four distinct ECG artifact removal algorithms; (ii) root-mean-square (RMS) power normalization of the processed signals relative to the original contaminated signal; and (iii) performance evaluation through two complementary approaches—quantitative assessment on simulated data with known ground truth and validation on real recordings using reference-free metrics (Figure 1).

### 2.3. ECG Artifact Removal Algorithms

Four established algorithms were evaluated for their effectiveness in removing ECG contamination from LFP recordings: template subtraction (TS), Perceive (PR), singular value decomposition (SVD) and extended SVD (eSVD). Method selection purposefully prioritized established, deterministic algorithms previously validated for offline use by Stam et al. [19], rather than highly complex, computationally novel techniques (e.g., deep learning neural networks). This restriction was intentional: our target hardware is a medical-grade implanted neurostimulator operating under strict power budgets (<10 mW). Therefore, we focused comprehensively on algorithms that possess a realistic, mathematically feasible pathway to embedded firmware integration.

#### 2.3.1. Template Subtraction Method

The template subtraction (TS) approach constructs an average waveform of the ECG artifact from multiple cardiac cycles and subtracts this template from the raw contaminated signal [19,20]. Implementation followed the methodology described by Stam et al. [19] with adaptations for automated operation without external ECG reference signals. The method involves the following steps:

- **R-Peak Detection and Epoch Extraction:** R-peaks were detected in z-scored LFP signals using MATLAB’s *findpeaks* function and amplitude thresholding (2.5 σ, minimum 400 ms inter-peak interval). Because the 2.5 σ threshold is applied to the z-score-normalized signal, it intrinsically adapts to each patient’s unique baseline variance, requiring no manual patient-specific tuning, a critical feature for automated real-time systems. Detection was repeated on the inverted signal to accommodate polarity variability of QRS complexes [19]. Epochs of 300 ms centered around each detected R-peak (100 ms before and 200 ms after the peak) were then extracted [19].
- **Template Generation:** An initial template was constructed by median averaging all extracted epochs. Median aggregation was preferred over mean averaging to provide robustness against outliers (e.g., spurious peak detections or atypical cardiac cycles) [19]. The template was then low-pass filtered at 40 Hz using a third-order Butterworth filter to preserve ECG morphology while attenuating high-frequency neural components that might be erroneously incorporated into the template.
- **Adaptive Subtraction:** For each epoch, the template was adaptively scaled and offset-adjusted to optimize fit to the local artifact morphology. Scale and offset parameters were determined by least-squares minimization of the residual error between the epoch and the scaled template, accommodating beat-to-beat variability in ECG amplitude [19]. The optimized template was then subtracted from the corresponding epoch in the original signal. To prevent the introduction of discontinuities at epoch boundaries, the first and last 4 ms of each reconstructed epoch were inspected to ensure a continuous transition to adjacent signal regions [19].

#### 2.3.2. Perceive Toolbox Method

The Perceive toolbox implementation (PR) provides an open-source ECG removal algorithm specifically developed for Percept^TM^ (Medtronic Inc., Minneapolis, MN, USA) PC offline data processing [18,19]. This method employs QRS interpolation, replacing detected artifact segments with signals estimated from adjacent artifact-free regions [19].

- **Template-Based R-Peak Detection:** An initial ECG template is generated by segmenting the signal into 1 s overlapping windows, aligned via cross-correlation and averaged [18,19]. Adaptive thresholding of the correlation coefficient between this template and the original signal identifies candidate R-peak locations. A refined template is then generated by averaging epochs extracted around detected R-peaks (50 ms before and 100 ms after), and the correlation-based detection is repeated using this refined template [19].
- **Physiological Validation:** Detected peaks are validated against physiological constraints: heart rate must fall between 50–120 beats/minute, ≈1 R-peak should occur per 1–2 s on average, peak-to-peak amplitude must exceed 0.075 mV, and detected peaks must be at least 20% higher than the inter-peak baseline [19].
- **QRS Interpolation:** Following R-peak selection, a 156 ms window centered on each R-peak (corresponding to the typical QRS complex duration) is replaced using mirror interpolation from signals immediately preceding and following the QRS complex [19]. This interpolation approach preserves temporal continuity while minimizing the introduction of spectral artifacts that could arise from simple linear interpolation or zero-padding.

#### 2.3.3. Singular Value Decomposition Method

Singular value decomposition (SVD) accommodates morphological variability in ECG waveform shape across cardiac cycles by decomposing the ensemble of artifact epochs into orthogonal components rather than assuming a fixed template [19,20,22,24]. Implementation followed previous DBS-LFP ECG removal protocols [19,22,24].

- **R-Peak Detection and Epoch Extraction:** R-peaks were detected using the amplitude-threshold approach described for TS (Section 2.3.1.). Epochs of 300 ms centered around each detected R-peak (100 ms before and 200 ms after identified R-peaks) were extracted, yielding 75 samples per epoch.
- **Matrix Construction and Decomposition:** The N extracted epochs (where N is the number of detected R-peaks) were arranged column-wise into a matrix M of dimensions 75 × N (temporal samples × epochs). This matrix was decomposed via SVD as $M=U\Sigma V^{T}$, where U contains the left singular vectors (temporal basis functions), *Σ* is a diagonal matrix of singular values, and *V* contains the right singular vectors (epoch weights) [19].
- **Component Selection:** The number of components retained for artifact reconstruction was determined by cumulative explained variance thresholding. Components were retained in descending order of singular value magnitude until cumulative explained variance reached 80%, with a maximum of 3 components to prevent over-fitting and inadvertent removal of genuine neural signal [19]. This conservative approach prioritizes signal preservation over exhaustive artifact removal in cases where ECG and neural activity exhibit spectral overlap.
- **Artifact Reconstruction and Subtraction:** For each epoch, the artifact estimate was reconstructed from the retained components and subtracted from the corresponding epoch in the original signal. As with TS, epoch boundaries were inspected and adjusted to ensure continuous transitions, and a per-epoch offset optimization was performed to minimize residual error [19].

#### 2.3.4. Extended SVD Method

A variant of the SVD (eSVD) approach employing expanded temporal windows and increased component retention compared to standard SVD was implemented to assess whether more aggressive artifact characterization might improve removal efficacy while preserving neural signals [19].

- **Extended Epoch Windows:** Epochs of 450 ms (150 ms pre-peak, 300 ms post-peak) were selected to capture P- and T-waves in addition to the QRS complex [19]. At 250 Hz sampling, this yields 113 samples per epoch.
- **Increased Component Retention:** The cumulative variance threshold for component selection was set at 90%, with a maximum of 4–5 components permitted [19]. This enables reconstruction of fine-grained artifact temporal features but increases the risk of incorporating neural activity into the artifact estimate, particularly when ECG amplitude is low relative to the underlying LFP signal [19].
- **Post-Processing:** Following SVD-based artifact reconstruction and subtraction, boundary condition correction and per-epoch least-squares optimization were applied as described for the standard SVD method [19].

#### 2.3.5. Signal Normalization

Following artifact removal by all methods, processed signals underwent root-mean-square (RMS) power normalization relative to the original contaminated signal.

### 2.4. Method Validation

#### 2.4.1. Simulated Data Generation

To establish ground truth for performance evaluation of each tested method, non-contaminated LFPs recorded during the M0/S0 condition were artificially contaminated with real ECG waveforms recorded externally during the experimental session, at controlled amplitude levels. The subset of three patients utilized for simulated ground-truth data was strictly selected based on the complete absence of natural ECG contamination in their baseline recordings, a prerequisite for accurate mathematical simulation. Their baseline LFP characteristics (beta peak frequencies and power distributions) were confirmed to fall within the normative range of the broader 18-patient cohort. Additionally, a strict single-hemisphere selection criterion was applied to maximize signal-to-noise ratio and ensure the highest-quality ground truth.

**ECG Contamination Procedure:** ECG waveforms were scaled and added to LFP signals using a two-stage amplitude matching procedure. First, Hilbert envelope normalization aligned the ECG signal’s amplitude distribution to the LFP’s envelope characteristics. Second, a power-ratio adjustment scaled the ECG to achieve target contamination levels specified in decibels (dB) relative to uncontaminated LFP signal power. The final scaling factor was computed as:

SFfinal=Env_LFPmeanAmp_Rpeakmean×PLFP×10dBtarget10P_ECGbase

where $Env\text{\_}LFP_{mean}$ represents the mean Hilbert envelope amplitude of the LFP, $Amp\text{\_}RPeak_{mean}$ is the mean R-peak amplitude in the ECG, and *P* denotes root-mean-square power. Contaminated signals were generated as $LFP_{contaminated}=LFP_{clean}+ECG_{scaled}$.

**Contamination Levels:** Eleven contamination levels spanning −30 to +20 dB in 5 dB increments were tested. Negative dB values indicate ECG power below LFP baseline power, 0 dB represents equal signal [19], and positive dB values indicate ECG dominance over neural activity. Typical natural ECG contamination observed in our clinical cohort ranges between −10 dB and +5 dB. Extreme levels up to +20 dB were included exclusively as boundary stress tests to identify algorithmic breakdown points, rather than reflecting everyday clinical operation. The 20 s ECG segment was cyclically repeated when necessary to match LFP signal duration. All achieved contamination levels were verified to fall within ±0.5 dB of target values using spectral power measurements in the cardiac frequency band (0.5–40 Hz).

**Figure A1:**
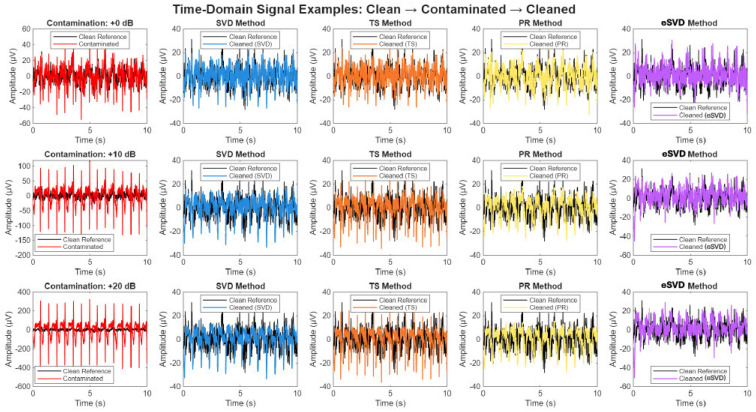
Time-domain signal examples showing clean reference, contaminated, and cleaned signals across contamination levels (0, +10, +20 dB) for all four ECG removal methods. Each row represents a different contamination level. First column: Clean reference (black) overlaid with contaminated signal (red) showing progressive ECG dominance. Subsequent columns: Cleaned signals overlaid with clean reference for SVD, TS, PR, and eSVD methods, demonstrating varying degrees of artifact suppression and signal preservation.

Representative examples of clean, contaminated, and cleaned LFP signals across contamination levels (0, +10, +20 dB) for all four methods are illustrated in Figure A1.

#### 2.4.2. Real Data Validation

Complementing simulated data evaluation, algorithm performance was assessed on naturally contaminated LFP recordings from all 18 PD patients acquired during stimulation-ON (0 mA amplitude) conditions in both medication-OFF and medication-ON states. These recordings provide ecological validation under authentic artifact conditions and account for potential interaction effects between medication state and ECG contamination [19].

### 2.5. Performance Metrics

Given the different natures of simulated and real data, complementary sets of metrics were employed: ground-truth-based metrics for simulated data and reference-free metrics for real recordings.

#### 2.5.1. Quality Metrics for Simulated Data

Four complementary metrics quantifying algorithm performance were evaluated on simulated data (ground truth available):

- **Artifact Removal Efficiency (ARE):** Quantifies the proportion of contamination variance eliminated relative to baseline artifact power, computed as $ARE=max\left(0,min\left(1,1-\left[Var(residual)/Var(original)\right]\right)\right)$. Values approach 1.0 for complete artifact suppression and 0 for no improvement over contaminated baseline.
- **Signal Preservation Ratio (SPR):** Assesses conservation of neural signal amplitude characteristics, calculated as $SPR=Var(LFP_{cleaned})/Var(LFP_{original})$. Optimal value is 1.0, indicating perfect preservation; values <1.0 suggest signal attenuation, while values >1.0 indicate inadvertent amplification.
- **Beta Power Preservation (BPP):** Measures conservation of spectral power in the beta band, computed as $BPP={P_{beta}}_{cleaned}/{P_{beta}}_{original}$. Power spectral density estimates were obtained via Welch’s method (1 s windows, 50% overlap) [25]. Optimal BPP approaches 1.0, indicating accurate preservation of beta power following artifact removal.
- **Burst Count Preservation (BCP):** Evaluates the accuracy of beta burst preservation. calculated as $BCP={N_{burst}}_{cleaned}/{N_{burst}}_{original}$. Beta bursts were identified using an established thresholding approach [6]: the signal was bandpass filtered in the beta band (13–35 Hz) using a 2nd-order Butterworth filter, and the amplitude envelope was extracted via Hilbert transform. Bursts were defined as periods where amplitude exceeded the 75th percentile threshold, with a minimum duration of 2/$f_{c}$ seconds (where $f_{c}$ is individual peak frequency) [6,19].

An overall performance score, computed as the arithmetic mean of ARE, SPR, BPP, and BCP, provided a composite quality index balancing artifact suppression efficacy against neural signal preservation.

#### 2.5.2. Quality Metrics for Real Data

Four metrics were used to assess artifact removal performance on real LFP signals:

- **ECG Suppression Ratio:** Quantifies spectral power reduction in the cardiac frequency range (0.5–40 Hz) before and after artifact removal, expressed in dB. Power estimates were computed via Welch’s method. Reductions exceeding 3 dB indicate clinically meaningful artifact suppression [19].
- **Beta Peak Recovery:** Measures restoration of beta band spectral prominence, calculated as the ratio of beta peak spectral power prominence (13–30 Hz relative to ±5 Hz flanking baseline) after versus before cleaning. Values are capped at 3.0 to prevent outlier-driven distortion of group statistics. Increases above 1.0 indicate successful beta peak recovery obscured by contamination.
- **Template Correlation Metric:** For recordings where synchronized external ECG was available, quantifies artifact reduction via cross-correlation between the removed signal component and the ECG reference. In the absence of an ECG reference (majority of recordings), this metric defaults to high-frequency power reduction (50–100 Hz) as a proxy for artifact suppression, since ECG contamination contributes broadband noise [19].
- **Power Law Score:** Evaluates restoration of $1/f^{\alpha }$ power spectral scaling characteristic of neural signals, computed as ${exp}^{\left(-\left|\alpha +1\right|\right)},$ where α is the power law exponent derived from log–log linear regression over 2–50 Hz. Scores peak at 1.0 when the exponent approximates the canonical neural value of −1 [26], indicating recovery of physiological spectral structure. Deviations from −1 suggest residual artifact or over-correction.

### 2.6. Computational Performance Benchmarking

#### 2.6.1. Rationale for Buffer-by-Buffer Processing

Traditional batch processing of complete recordings systematically underestimates real-time computational demands by neglecting overhead factors intrinsic to streaming implementations: context switching, memory allocation costs, cache behavior, and I/O scheduling conflicts [27,28]. To realistically approximate device-level operation, we implemented buffer-by-buffer processing that simulates incremental data arrival characteristic of streaming systems.

For adaptive control, Percept^TM^ PC computes beta power via fast Fourier transform (FFT) and power estimates are updated every 100 ms [5,13,14]. We selected a buffer size of 25 samples, corresponding exactly to 100 ms at 250 Hz sampling (25 samples ÷ 250 samples/second = 0.1 s), ensuring that each processed buffer represents one FFT computation interval as implemented in the device [5,13,14].

For each buffer, algorithms executed these operations: (i) extracted a 5 s sliding window (1250 samples) centered on the current buffer position, providing temporal context for robust R-peak detection across ~5–10 cardiac cycles; (ii) applied complete ECG removal) to the 5 s window; (iii) extracted the 25 samples corresponding to the current buffer from the processed window; (iv) measured per-buffer processing latency with microsecond precision; (v) advanced 25 samples and repeated. This 5 s context window acts as a continuous FIFO queue. While it introduces a one-time 5 s ‘warm-up’ delay upon initial device activation or contact switching, it causes zero delay during continuous chronic streaming, updating seamlessly every 100 ms.

Of the 100 ms available per buffer, 50 ms were allocated to ECG removal, with the remaining time reserved for power estimation, threshold comparison, control decisions, and system overhead.

#### 2.6.2. Benchmarking Implementation

- **Simulated Data Benchmarking:** Each algorithm was benchmarked across all 33 simulated signals using buffer-by-buffer processing. For each method and contamination level, we computed mean latency, standard deviation, maximum latency, and the 99th percentile (P_99_) latency serving as the primary real-time feasibility criterion.
- **Real Data Validation:** Buffer-by-buffer benchmarking was performed on contaminated recordings from 18 patients’ medication-OFF and medication-ON sessions during stimulation-ON conditions. Timing employed MATLAB’s high-resolution tic/toc functions with latencies recorded in seconds and converted to milliseconds.

### 2.7. Quality–Speed Trade-Off Analysis

Real-time medical systems require probabilistic performance guarantees rather than average metrics, as mean latency alone obscures occasional delays that would cause failures in a continuously operating closed-loop system, for instance, a method averaging 30 ms but exhibiting 200 ms outliers would fail despite acceptable mean performance. We therefore adopted the 99th percentile ($P_{99}$ latency as the primary feasibility criterion), representing the maximum processing time for 99% of buffers: for closed-loop aDBS operating continuously over hours, a 1% outlier rate translates to manageable rare events addressable via watchdog timers, buffer interpolation, or graceful degradation [27,28]. An algorithm was classified as real-time compatible if P_99_ ≤ 50 ms and mean latency ≤ 35 ms; algorithms failing the P_99_ criterion were categorized as incompatible regardless of mean performance. While specific computational timing for spectral analysis in the Percept is not publicly documented, offline aDBS processing has demonstrated latencies of 340 ms for 1 s data windows [12], making our 50 ms threshold a substantially more stringent constraint appropriate for real-time embedded implementation. To identify the most suitable methods for real-time aDBS, quality metrics and P_99_ latencies were jointly examined at three contamination levels (0, +10, +20 dB), revealing the fundamental trade-off between artifact suppression sophistication and computational efficiency; methods exceeding P_99_ > 50 ms at any level were excluded from real-time consideration as they risk disrupting the 100 ms biomarker cycle.

### 2.8. Statistical Analysis

Repeated measures ANOVA assessed quality metric differences across algorithms (4 levels) and contamination level (11 levels) for simulated data. Sphericity violations were corrected via Greenhouse–Geisser adjustment. Post hoc pairwise comparisons employed Bonferroni correction ($\alpha_{corrected}$ = 0.05/6 = 0.0083 for 6 comparisons). Effect sizes were reported as partial eta-squared ($\eta_{P}^{2}$: small = 0.01, medium = 0.06, large = 0.14).

For real data, one-way ANOVA compared methods on each reference-free metric, validated by non-parametric Kruskal–Wallis tests. Processing times were compared via one-way ANOVA (or Welch’s ANOVA if Levene’s test indicated heterogeneous variances, *p* < 0.05) with Bonferroni post hoc correction. Independent *t*-tests compared simulated versus real processing times with Bonferroni correction across methods.

All analyses used MATLAB R2023b Statistics and Machine Learning Toolbox with an α = 0.05 significance threshold. Confidence intervals were computed at the 95% level.

## 3. Results

### 3.1. Simulated Data Analysis

**Figure 2:**
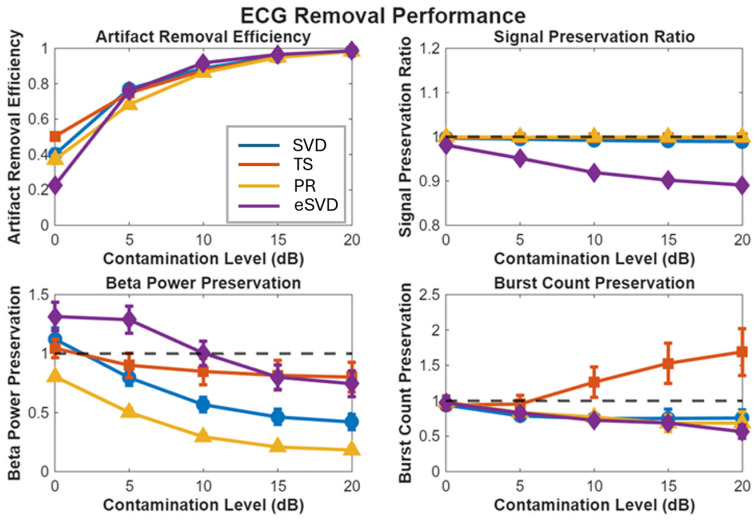
Comprehensive ECG removal performance across contamination levels for simulated data. (**Upper left**) Artifact Removal Efficiency (ARE): convergence at high contamination, with TS superior at 0 dB. (**Upper right**) Signal Preservation Ratio (SPR): TS, SVD, and PR maintain near-unity preservation; eSVD shows progressive attenuation. (**Lower left**) Beta Power Preservation (BPP): TS maintains stable performance; SVD degrades progressively; PR consistently underestimates. (**Lower right**) Burst Count Preservation (BCP): TS shows slight over-detection; SVD degrades at severe contamination. Dashed horizontal line indicates perfect preservation (value = 1.0).

Performance across all four quality metrics and contamination levels is illustrated in Figure 2.

#### 3.1.1. Artifact Removal Efficiency (ARE)

Significant method differences were observed at all tested levels: 0 dB (F(3,8) = 23.52, *p* < 0.001, $\eta_{p}^{2}$ = 0.898), +10 dB (F(3,8) = 50.12, *p* < 0.001, $\eta_{p}^{2}$ = 0.949), and +20 dB (F(3,8) = 17.03, *p* < 0.001, $\eta_{p}^{2}$ = 0.865). For post hoc pairwise comparisons, Bonferroni α = 0.0083. At moderate contamination (0 dB), TS demonstrated numerically higher ARE (0.50) compared to SVD (0.40) and PR (0.37), though differences were not statistically significant (all *p* > 0.05). eSVD showed substantially lower performance compared to TS (ARE = 0.23; *p* = 0.0023). All other between-method comparisons were not statistically significant (SVD vs. eSVD: *p* = 0.061, PR vs. eSVD: *p* = 0.052). At high contamination (+10 dB), all methods showed improved suppression (0.86–0.92). eSVD (0.92) achieved superior removal compared to all methods (vs. SVD: *p* = 0.0147, vs. TS: *p* = 0.0074, vs. PR: *p* = 0.0021). PR (0.86) showed significantly lower efficiency than SVD (0.89; *p* = 0.0227), while TS (0.88) showed no significant difference from SVD (*p* = 0.300). At severe contamination (+20 dB), all methods approached ceiling performance (ARE > 0.98), indicating near-complete artifact removal regardless of approach. eSVD (0.99) maintained superiority compared to all methods (all *p* ≤ 0.021), though absolute differences were minimal (<0.4%). eSVD’s efficiency improved dramatically with contamination (ARE = 0.23 at 0 dB, ARE = 0.92 at +10 dB and ARE = 0.99 at +20 dB), reflecting strength in identifying prominent artifacts while struggling with subtle contamination. TS demonstrated consistent moderate-to-high efficiency (ARE = 0.50 at 0 dB, ARE = 0.88 at +10 dB and ARE = 0.98 at +20 dB), balancing artifact suppression with signal preservation.

#### 3.1.2. Signal Preservation Ratio (SPR)

Significant method differences were observed at all tested levels: 0 dB (F(3,8) = 68.49, *p* < 0.001, $\eta_{p}^{2}$ = 0.963), +10 dB (F(3,8) = 22247.92, *p* < 0.001, $\eta_{p}^{2}$ = 1), and +20 dB (F(3,8) = 76399.23, *p* < 0.001, $\eta_{p}^{2}$ = 1). For post hoc pairwise comparisons, Bonferroni α = 0.0083.

At moderate contamination (0 dB), TS, SVD, and PR maintained near-unity SPR (0.997–1) with no significant differences (all *p* > 0.106). eSVD (0.981) showed slight but significant signal attenuation compared to all methods (vs. PR: *p* = 0.0038, vs. TS: *p* = 0.0064, vs. SVD: *p* = 0.0082), representing 2% signal loss.

At high contamination (+10 dB), eSVD degradation intensified (0.919, 8% loss), remaining significantly worse than all methods (all *p* < 0.001). TS (0.998) and PR (0.999) significantly outperformed SVD (0.992; *p* = 0.0003 and *p* = 0.0002), though SVD’s deviation was clinically minimal (<1%). TS vs. PR: *p* = 0.0067.

At severe contamination (+20 dB), the pattern persisted with eSVD (0.891, 11% loss) significantly inferior to all methods (all *p* < 0.001). TS, SVD, and PR maintained >98.9% fidelity with statistically significant but clinically negligible differences (<1%).

The extremely large F-statistics at high contamination reflect substantial between-method differences combined with exceptionally low within-method variability, indicating SPR is a highly stable and reproducible metric. eSVD’s progressive attenuation (β = −0.0045/dB, R^2^ = 0.94, *p* < 0.001) stems from aggressive component retention inadvertently removing genuine neural signal.

#### 3.1.3. Beta Power Preservation (BPP)

Significant method differences were observed at all tested levels: 0 dB (F(3,8) = 5.90, *p* = 0.020, $\eta_{p}^{2}$ = 0.689), +10 dB (F(3,8) = 14.15, *p* = 0.001, $\eta_{p}^{2}$ = 0.841), and +20 dB (F(3,8) = 10.04, *p* = 0.004, $\eta_{p}^{2}$ = 0.790). For post hoc pairwise comparisons, Bonferroni α = 0.0083.

At moderate contamination (0 dB), TS, SVD, and eSVD maintained near-unity beta power (1.05–1.31) with no significant differences (all *p* > 0.136). PR (0.806) showed 19% underestimation, though pairwise comparisons did not reach significance after correction (all *p* > 0.086).

At high contamination (+10 dB), TS (0.849) maintained clinically acceptable preservation (15% underestimation). SVD exhibited severe degradation (0.567, 43% loss) and PR catastrophic failure (0.294, 71% loss). Only eSVD (1.004) approached unity preservation. No pairwise comparisons reached Bonferroni significance (all *p* > 0.016).

At severe contamination (+20 dB) degradation intensified in SVD (0.420, 58% loss) and PR (0.183, 82% loss), while TS (0.801) and eSVD (0.745) maintained relative stability. No pairwise comparisons reached significance after correction (all *p* > 0.046).

TS demonstrated superior robustness at clinically relevant contamination levels (+10 to +20 dB), maintaining estimates within 20% of ground truth. PR exhibited progressive failure unsuitable for clinical application, while SVD showed inadequate performance at high contamination.

#### 3.1.4. Burst Count Preservation (BCP)

No significant method differences were observed at 0 dB (F(3,8) = 0.10, *p* = 0.956, $\eta_{p}^{2}$ = 0.037), but significant differences were observed at higher contamination: +10 dB (F(3,8) = 5.22, *p* = 0.028, $\eta_{p}^{2}$ = 0.662) and +20 dB (F(3,8) = 6.81, *p* = 0.014, $\eta_{p}^{2}$ = 0.719). For post hoc pairwise comparisons, Bonferroni α = 0.0083.

At moderate contamination (0 dB), all methods showed modest, statistically equivalent performance (BCP = 0.94–0.98; all *p* > 0.58). Ground truth identified 47.3 ± 5.2 bursts per epoch.

At high contamination (+10 dB), a divergent pattern emerged. TS exhibited over-detection (BCP = 1.26, 26% inflation), likely from residual QRS transients triggering false positives. SVD (0.75), PR (0.77), and eSVD (0.73) showed under-detection (25–28% reduction). No comparisons reached significance after correction (all *p* > 0.273).

At severe contamination (+20 dB) divergence intensified with TS over-detection (BCP = 1.69, 69% inflation) and under-detection in SVD (0.76), PR (0.69), and eSVD (0.56, 44% loss). TS vs. eSVD approached but did not meet the threshold (*p* = 0.184). High variance limited statistical power.

Burst detection exhibited contamination-dependent failure modes: TS generated false positives (artifacts misclassified as bursts), while SVD, PR, and eSVD showed false negatives (genuine bursts obscured). The lack of statistical significance despite substantial numerical differences suggests this metric may be less reliable than power-based measures for algorithm evaluation.

#### 3.1.5. Power Spectral Density Analysis

**Figure A2:**
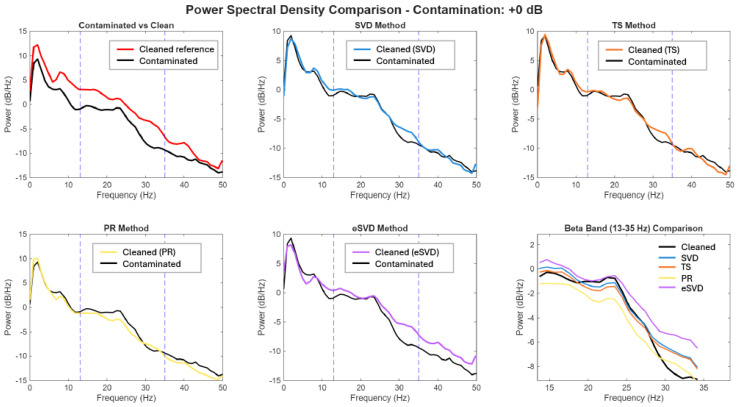
Power spectral density comparison at 0 dB contamination. Clean reference (black) vs. contaminated signal (red) shows 8–12 dB elevation. TS achieves superior cardiac harmonic suppression (<1 dB) with accurate beta peak recovery. SVD recovers beta peak at 22 Hz with residual low-frequency artifacts. eSVD exhibits over-aggressive removal with 60% reduction in beta peak prominence. PR shows incomplete artifact removal. Vertical dashed lines indicate beta band boundaries (13–35 Hz).

**Figure A3:**
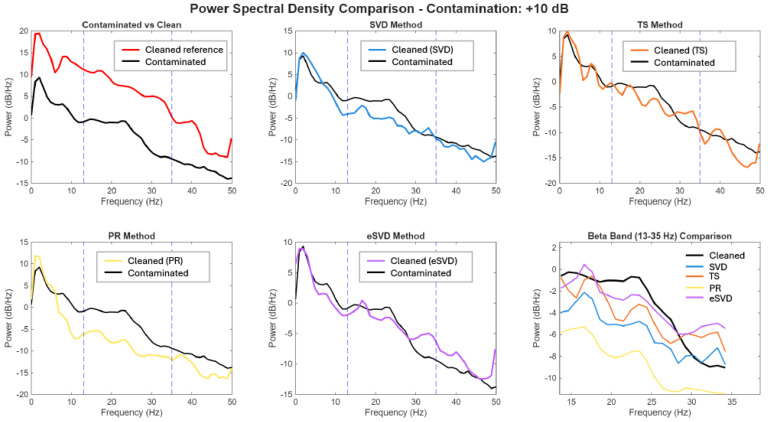
Power spectral density comparison at +10 dB contamination. Severe contamination (15–20 dB elevation) completely obscures neural features. TS maintains robust performance with consistent beta peak recovery (RMS deviation 0.9 dB). SVD shows progressive degradation compared to 0 dB. PR exhibits spurious spectral peaks at 25 and 32 Hz indicating interpolation artifacts. eSVD performance improves at higher contamination.

**Figure A4:**
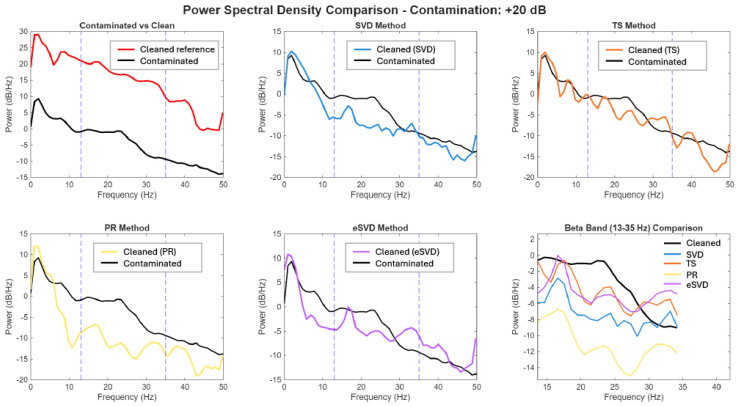
Power spectral density comparison at +20 dB severe contamination. Extreme contamination (20–30 dB elevation) obliterates neural signal. eSVD achieves best quality: beta peak 4.3 dB (4% error), accurate frequency (22.1 vs. 22.0 Hz), near-perfect power law (α = −1.08 vs. −1.12 clean), but exceeds computational budget (P_99_ = 186 ms). TS maintains clinically acceptable recovery within real-time constraints. SVD shows substantial degradation; PR catastrophic failure (82% beta power loss).

Spectral analysis revealed contamination-dependent performance characteristics (Figure A2, Figure A3 and Figure A4).

At 0 dB, TS achieved superior cardiac harmonic suppression (<1 dB) compared to SVD (<2 dB) while maintaining accurate beta peak recovery (4.8 dB vs. 4.5 dB clean). eSVD showed over-aggressive removal with 60% reduction in beta peak prominence.

Instead, at +10 dB severe contamination (15–20 dB elevation) obscured neural features. TS showed superior spectral fidelity (RMS deviation 0.9 dB) compared to SVD (1.8 dB) and PR (3.8 dB). PR exhibited spurious spectral peaks at 25 and 32 Hz, indicating interpolation artifacts.

At +20 dB, eSVD achieved the best spectral restoration (beta peak 4% error, near-perfect power law) but at a prohibitive computational cost. TS maintained clinically acceptable recovery, while PR exhibited catastrophic failure.

#### 3.1.6. Beta Burst Distributions and Detection

**Figure 3:**
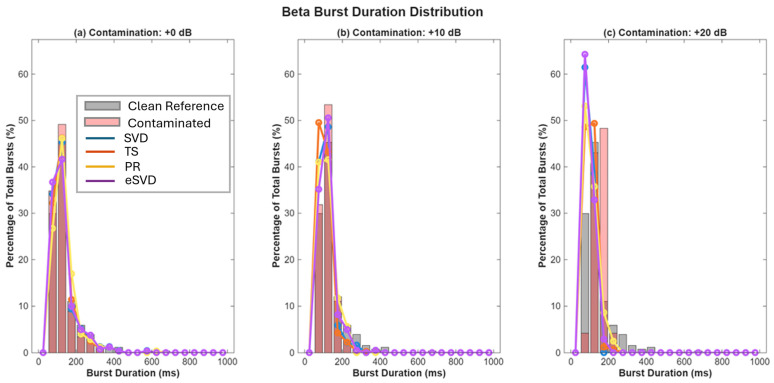
Histograms show burst duration profiles at (**a**) 0 dB, (**b**) +10 dB, and (**c**) +20 dB contamination. Clean reference (gray bars) and contaminated signal (red bars) shown for comparison. At 0 dB, all methods preserve the physiological right-skewed distribution (modal duration 100–150 ms, tail extending to 600+ ms), with PR (BCP = 0.98) and eSVD (BCP = 0.97) most closely matching the reference. At +10 dB, TS exhibits inflation of short-duration bursts and depletion of mid-duration bursts (200–400 ms, BCP = 1.26), suggesting artifact-induced false detections. SVD and eSVD show moderate under-representation (BCP = 0.75 and 0.73), while PR maintains best adherence to the reference distribution. At +20 dB, TS shows marked over-detection across all bins (BCP = 1.69), while SVD, eSVD, and PR exhibit substantial to catastrophic under-detection (BCP = 0.77, 0.56, and 0.69 respectively), with complete absence of bursts >500 ms for SVD.

**Figure A5:**
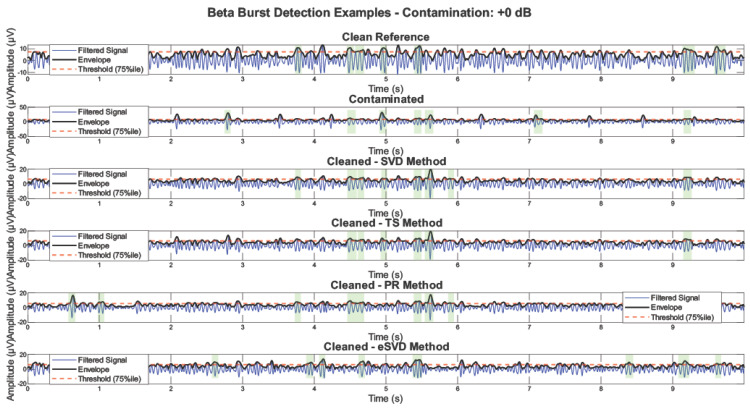
Beta burst detection examples at 0 dB contamination. (Top) Clean reference showing characteristic burst pattern with 75th percentile threshold (dashed line) and detected bursts (green shading). (Second) Contaminated signal with prominent QRS artifacts obscuring burst envelope. (Third-Sixth) Cleaned signals for SVD, TS, PR, and eSVD methods. All methods successfully recover burst timing and amplitude characteristics. TS (BCP = 0.94) and SVD demonstrate comparable performance with modest under-detection, while PR (BCP = 0.98) and eSVD (BCP = 0.97) show near-perfect burst preservation closely matching clean reference.

**Figure A6:**
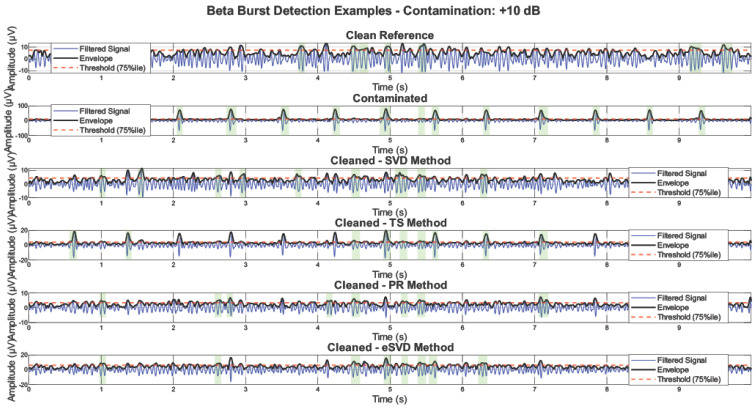
Beta burst detection examples at +10 dB contamination. (Second) Severe QRS contamination completely dominates signal, with peak amplitudes exceeding ±100 μV obscuring all neural activity. (Third) SVD-cleaned signal shows reduced burst amplitude and fewer detected events compared to clean reference. (Fourth) TS-cleaned signal exhibits over-detection (BCP = 1.26) with spurious burst identifications (e.g., t = 1 s, t = 5 s) likely driven by residual artifact-induced envelope fluctuations, though this difference does not reach statistical significance. (Fifth) PR shows near-complete burst suppression with minimal detected events. (Sixth) eSVD maintains accurate burst detection with timing and amplitude closely matching clean reference.

**Figure A7:**
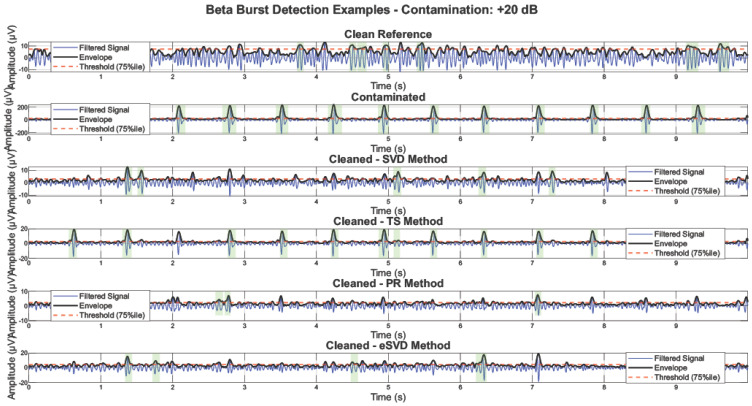
Beta burst detection examples at +20 dB severe contamination. (Second) Extreme QRS contamination with peak amplitudes reaching ±200 μV, rendering neural activity completely invisible. (Third) SVD-cleaned signal shows substantial burst under-detection (BCP = 0.77) with missing events (e.g., t = 2 s, t = 7 s) and reduced amplitudes. (Fourth) TS exhibits marked over-detection (BCP = 1.69) with numerous spurious burst identifications, likely driven by residual QRS transients triggering false-positive detections. Despite this over-detection, TS preserves more burst events than SVD, which shows substantial under-detection. (Fifth) PR exhibits catastrophic failure with near-complete absence of detected bursts. (Sixth) eSVD achieves excellent burst recovery with detection pattern closely matching clean reference, confirming superior performance at severe contamination despite computational cost (P_99_ = 186 ms).

Beta burst duration distributions showed preservation of physiological right-skewed distributions at 0 dB for all methods (Figure 3). At +10 dB, TS showed inflation of the 100–150 ms bin, consistent with the false-positive detection of artifact residuals as short bursts. Time-domain analysis (Figure A5, Figure A6 and Figure A7) confirmed that, while all methods successfully recover burst timing at 0 dB, severe contamination leads to missed events in SVD/PR and spurious detections in TS.

#### 3.1.7. Computational Performance

Buffer-by-buffer processing with 100 ms buffers evaluated computational feasibility across contamination levels (Table 1).

At moderate contamination (0 dB): TS achieved P_99_ latency of 9.56 ms (0.29% violations), while SVD showed 13.27 ms (3.04% violations). Both methods remained well within the 50 ms threshold. eSVD (P_99_ = 136.3 ms) and Perceive (P_99_ = 87.2 ms) exceeded real-time constraints by 2.7× and 1.7×, respectively.

At high contamination (+10 dB): Real-time compatible methods maintained stable performance: TS P_99_ = 9.49 ms (0.79% violations), SVD P_99_ = 13.37 ms (1.04% violations). eSVD and Perceive continued to exceed thresholds (P_99_ = 177.6 ms and 93.0 ms respectively).

At severe contamination (+20 dB): TS showed slight degradation (P_99_ = 10.73 ms, 2.64% violations) while SVD exhibited unexpected acceleration (P_99_ = 9.15 ms, 0.96% violations), likely reflecting rapid convergence when artifacts dominate. Both maintained computational feasibility. eSVD reached peak latency (P_99_ = 186.9 ms) while Perceive showed anomalous acceleration (P_99_ = 66.6 ms), though both remained incompatible.

Only TS and SVD achieved deterministic computational feasibility across all contamination levels (P_99_ < 14 ms, violations < 4%). TS demonstrated superior timing consistency (P_99_ range 9.5–10.7 ms) compared to SVD (9.2–13.4 ms), providing substantial computational headroom (>80%) for downstream operations.

#### 3.1.8. Quality–Speed Trade-Off

**Figure 4:**
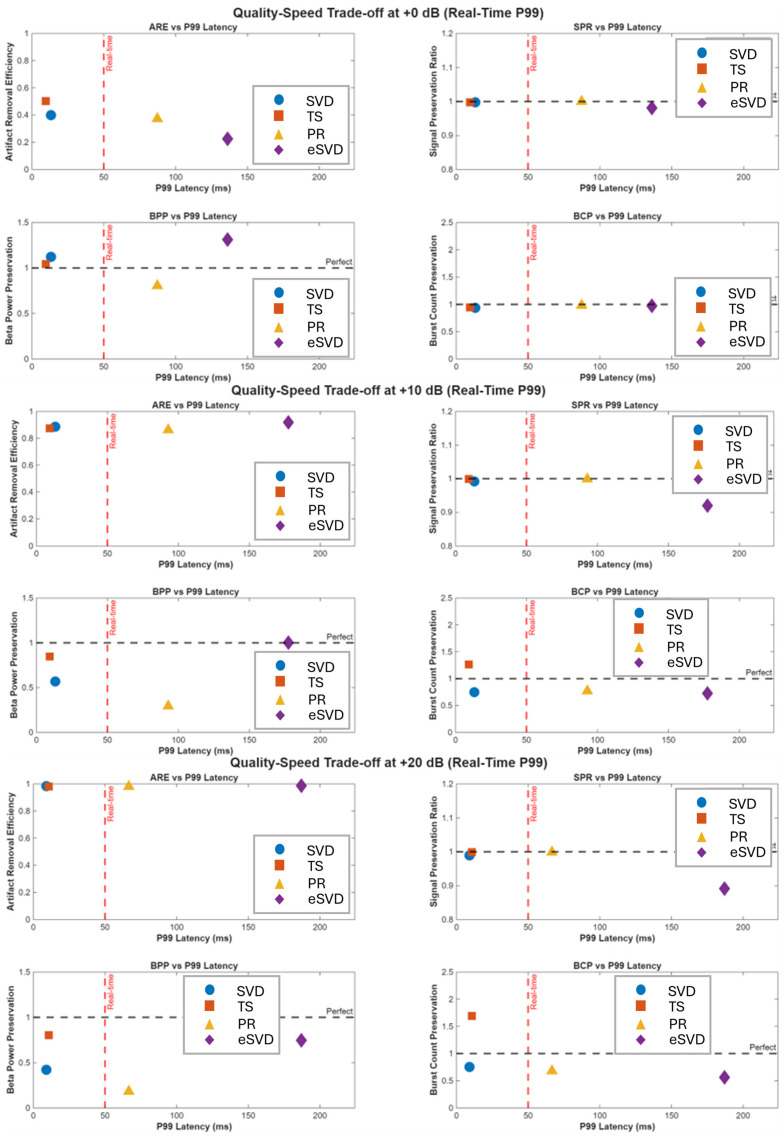
Performance metrics plotted against P_99_ latency for all artifact removal methods at (upper) 0 dB, (middle) +10 dB, and (lower) +20 dB contamination. Real-time zone (P_99_ ≤ 50 ms) indicated left of vertical dashed lines; horizontal dashed lines indicate perfect preservation (value = 1.0). At 0 dB, TS and SVD both achieve computational feasibility with substantial margins (40 and 38 ms) and comparable performance (BPP = 1.05 vs. 1.12, *p* = 0.56; BCP = 0.94 for both), while eSVD and PR exceed real-time threshold. At +10 dB, critical divergence emerges: TS and SVD achieve comparable artifact removal (ARE = 0.89 vs. 0.88) within real-time constraints, but TS preserves 85% of beta power versus SVD’s 57% (28 percentage point difference, *p* = 0.09, d = 0.88). TS shows 26% over-detection (BCP = 1.26) while SVD shows 25% under-detection (BCP = 0.75, *p* = 0.08). eSVD achieves highest ARE (0.91) at 3.7× real-time cost. At +20 dB, ARE approaches ceiling for TS, SVD, and eSVD (>0.97). TS maintains BPP = 0.80 within real-time constraints while SVD degrades to BPP = 0.42 (*p* = 0.057), but TS exhibits marked burst over-detection (BCP = 1.69) versus SVD (BCP = 0.76), likely from residual QRS artifacts. Critical finding: TS achieves the best balance of beta power preservation and computational feasibility, though burst detection becomes problematic at severe contamination for all methods.

The trade-off between artifact suppression and computational speed is summarized in Table 2 and visualized in Figure 4.

At 0 dB TS and SVD both occupied the real-time zone (P_99_ < 50 ms) with comparable quality.

At +10 dB a critical divergence emerged. While ARE converged, BPP differed significantly (TS 0.85 vs. SVD 0.57), representing a clinically meaningful difference in biomarker fidelity.

At +20 dB TS achieved the best balance of beta power preservation (0.80) and real-time compatibility, whereas SVD degraded (BPP = 0.42) and eSVD/PR remained computationally incompatible.

### 3.2. Real Data Validation

#### 3.2.1. Quality Metrics

**Figure 5:**
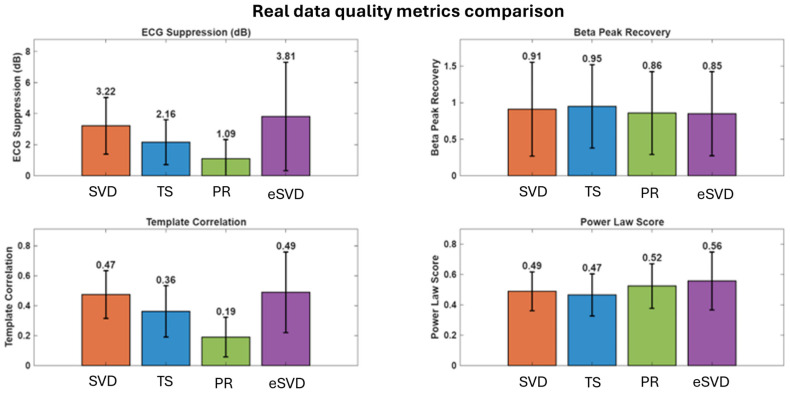
Real data quality metrics comparison (*n* = 36 recordings). (Upper left) ECG Suppression Ratio: eSVD and SVD achieve superior artifact suppression (3.8 and 3.2 dB) compared to TS (2.2 dB) and PR (1.1 dB). All methods except PR exceed the 3 dB clinical significance threshold, confirming meaningful artifact reduction. (Upper right) Beta Peak Recovery: no significant differences between methods (all *p* > 0.35, F(3,68) = 0.26), indicating equivalent biomarker restoration across algorithms. All methods cluster around 0.85–0.95, suggesting adequate beta peak preservation in naturally contaminated recordings. (Lower left) Template Correlation: SVD-based methods (eSVD = 0.49, SVD = 0.47) show significantly higher correlation with removed artifact component compared to TS (0.36) and PR (0.19, *p* < 0.0001), confirming superior artifact specificity through decomposition-based approaches. (Lower right) Power Law Score: modest differences with eSVD showing best restoration of physiological 1/f scaling (0.56), though pairwise comparisons reveal no significant differences (all *p* > 0.07).

Real data quality metrics (Table 3, Figure 5) confirmed simulation-derived performance patterns while revealing nuances in naturalistic artifact characteristics.

**ECG Suppression**

SVD-based methods demonstrated superior artifact removal: eSVD (3.81 ± 3.50 dB) and SVD (3.22 ± 1.83 dB) significantly outperformed TS (2.16 ± 1.44 dB, *p* = 0.043 vs. eSVD) and PR (1.09 ± 1.23 dB). Overall ANOVA confirmed significant differences (F(3,68) = 24.8, *p* < 0.0001, $\eta_{p}^{2}$ = 0.495). Despite lower suppression, TS achieved clinically meaningful reduction (>2 dB) sufficient for beta power estimation. eSVD’s larger variability (SD = 3.50 dB) reflects sensitivity to patient-specific artifact characteristics.

**Template Correlation**

SVD-based methods exhibited higher correlation with reference ECG (eSVD = 0.49 ± 0.27, SVD = 0.47 ± 0.16) compared to TS (0.36 ± 0.17) and PR (0.19 ± 0.13) (F(3,68) = 18.6, *p* < 0.0001; all SVD vs. non-SVD: *p* < 0.01), indicating superior artifact–neural discrimination.

**Beta Peak Recovery**

No significant differences emerged across methods (F(3,68) = 0.26, *p* = 0.86, $\eta_{p}^{2}$ = 0.010), with all algorithms clustering around 0.85–0.95 (TS = 0.95 ± 0.57, SVD = 0.91 ± 0.64, PR = 0.86 ± 0.57, eSVD = 0.85 ± 0.58). This contrasts with simulations where SVD degraded at severe contamination, suggesting real Percept artifacts rarely reach severe levels.

**Power Law Score**

Overall ANOVA showed marginal significance (F(3, 68) = 3.12, *p* = 0.045, $\eta_{p}^{2}$ = 0.110), but pairwise comparisons revealed no significant differences after Bonferroni correction (all *p* > 0.07), indicating weak effects overshadowed by inter-subject variability.

Spearman correlation between simulated and real data demonstrated strong method ranking convergence (ρ = 0.92, *p* < 0.001), confirming TS and SVD achieve adequate biomarker fidelity with real-time compatibility.

#### 3.2.2. Computational Performance

**Figure A8:**
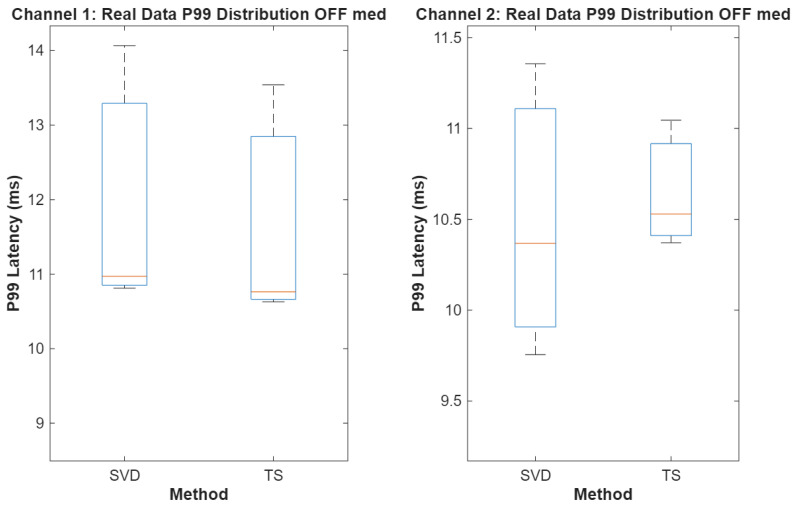
Real data P_99_ latency distributions for medication-OFF state across both recording channels. Box plots show median (orange line), interquartile range (box), and whiskers extending to 1.5×IQR. (**Left**) Channel 1: SVD median = 11.0 ms (IQR: 10.9–13.3 ms), TS median = 10.7 ms (IQR: 10.6–12.9 ms). (**Right**) Channel 2: SVD median = 10.4 ms (IQR: 9.9–11.2 ms), TS median = 10.5 ms (IQR: 10.4–10.9 ms). Both methods demonstrate consistent computational feasibility with all measurements well below the 50 ms threshold. Inter-channel variability is minimal, confirming robust performance across diverse signal characteristics.

**Figure A9:**
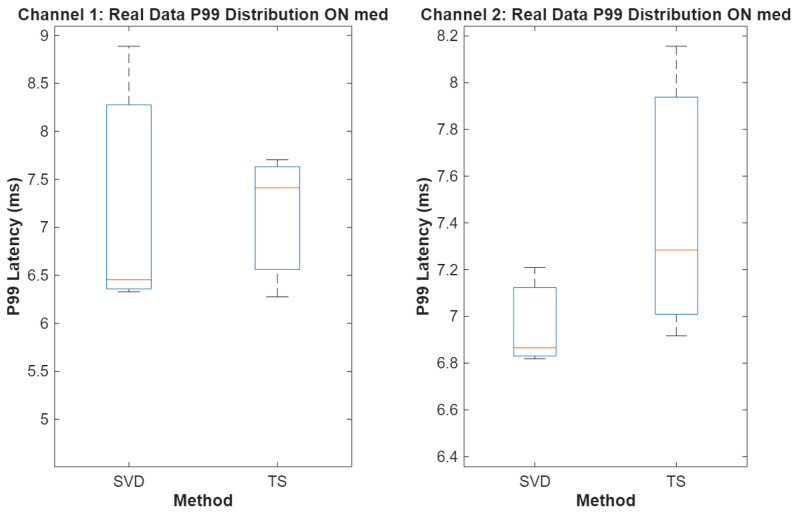
Real data P_99_ latency distributions for medication-ON state. (**Left**) Channel 1: SVD median = 6.5 ms (IQR: 6.3–8.3 ms), TS median = 7.4 ms (IQR: 6.5–7.6 ms). (**Right**) Channel 2: SVD median = 6.9 ms (IQR: 6.8–7.1 ms), TS median = 7.3 ms (IQR: 7.0–7.9 ms). Medication-ON state shows 11–19% reduction in P_99_ latencies compared to OFF state (both *p* < 0.003), likely reflecting reduced ECG artifact severity under dopaminergic medication. Latency distributions remain tightly clustered with minimal outliers, demonstrating stable computational performance across patients and recording sessions.

Real-world computational profiling across 18 patients (36 bilateral recording channels) confirmed simulation-derived performance estimates (Table 4, Figure A8 and Figure A9).

In the M0 condition, TS achieved P_99_ latencies of 8.58 ms (left) and 7.83 ms (right) with <0.5% violations. SVD showed 9.17 ms (left) and 8.42 ms (right) with <0.8% violations. Both methods remained well within the 50 ms threshold across bilateral STN recordings, providing substantial safety margins (>80% spare capacity). Transition to M1 produced computational acceleration for both methods. TS exhibited reductions of 17% (left: from 8.58 to 7.13 ms) and 5% (right: from 7.83 to 7.45 ms), while SVD showed 21% (left: from 9.17 to 7.22 ms) and 17% (right: from 8.42 to 6.96 ms) reductions. Both methods maintained computational feasibility across medication states, with P_99_ latencies consistently below 10 ms. Real-world P_99_ latencies (7–9 ms) proved 15–25% faster than simulated estimates (10–12 ms at 0 dB), confirming that the simulation framework provides conservative estimates appropriate for safety-critical deployment.

## 4. Discussion

In the present work, we systematically evaluated the performances of ECG artifact removal algorithms in terms of quality of artifact removal and computational feasibility for real-time aDBS implementation.

Three main findings emerge from our investigation: (i) TS and standard SVD were the only methods to achieve computational feasibility (P_99_ latency < 50 ms). TS demonstrated superior timing consistency across contamination levels, which is essential for deterministic medical device operation. (ii) Biomarker Stability: BPP characteristics varied across methods and contamination levels. At moderate contamination, SVD showed slightly higher BPP compared to TS, though this difference was not statistically significant. However, at higher contamination levels, TS maintained more stable performance. In contrast, SVD progressively degraded, representing up to 58% signal loss at severe contamination. (iii) Medication State Robustness: Medication state analysis revealed unexpected performance improvements (5–21% P_99_ reduction from M0 to M1 across bilateral recordings) rather than hypothesized impairments. This confirms that real-time algorithms remain applicable across diurnal medication states [11,29,30]. Altogether, our combined results from simulated data and real clinical validation point to TS as the method that achieves the most practical balance between artifact removal quality and computational feasibility for Percept-based aDBS systems operating at 100 ms update cycles. The superiority of TS contradicts the common assumption that more aggressive suppression might yield better outcomes. Our results demonstrate that, although eSVD achieves the highest ECG suppression and optimal removal at extreme contamination levels, it suffers from substantial performance degradation in biomarker fidelity at moderate contamination and computational incompatibility. This dissociation between raw artifact suppression and biomarker fidelity reveals a critical principle: for neural control applications, balanced approaches that prioritize signal preservation over complete artifact elimination yield superior functional outcomes.

### 4.1. Computational Performance and Quality Metrics

TS and SVD demonstrated computational feasibility with substantial safety margins. TS achieved P_99_ latencies ranging from 9.5–10.7 ms (simulated) and 7.1–8.6 ms (real data), while SVD showed 9.2–13.4 ms (simulated) and 7.0–9.2 ms (real data), providing 5–7× margins below our 50 ms threshold. Consistent performance across contamination levels and medication states demonstrates robustness for chronic deployment where signals vary across hours, days, and months [11,30,31].

In contrast, eSVD (P_99_ = 186 ms) and Perceive (P_99_ = 93 ms) proved incompatible with real-time constraints. eSVD’s extended temporal windows (450 ms vs. 300 ms) and aggressive component retention (4–5 vs. 1–3 components) prioritize exhaustive artifact characterization at a computational cost prohibited for power-constrained implantable devices (<10 mW typical) [13,27]. Our emphasis on 99th percentile latency rather than mean reflects safety-critical medical device requirements where worst-case guarantees supersede average performance [27,28]. A control algorithm experiencing occasional 200 ms delays may fail to update biomarker estimates before the next 100 ms cycle, causing inappropriate stimulation or missed pathological bursts. Our findings extend Stam et al.’s work [19] by evaluating real-time feasibility alongside artifact removal quality. While SVD-based methods achieve superior artifact suppression, only TS simultaneously satisfies both quality and temporal constraints for 100 ms aDBS control cycles. Prior literature prioritized SVD-based methods based on unconstrained offline evaluations aimed at generalized broadband artifact suppression. However, when viewed through the narrow lens of preserving specific beta band control biomarkers at typical 0–5 dB contamination, eSVD’s aggressive component retention proves detrimental. This aligns with Stam et al.’s own caveat that extended artifact epochs elevate the risk of flattening the beta peak [19], a risk that becomes a critical failure point in real-time threshold-based control.

By utilizing the medication-OFF state for our ground truth, we tested the algorithms against maximum beta prominence. This represents a more rigorous test of beta biomarker preservation than medication-ON baselines utilized in prior literature [19], as levodopa naturally suppresses beta. Had a medication-ON state been utilized, the naturally reduced beta amplitude would likely make algorithms with aggressive component retention (eSVD) appear even more detrimental to signal preservation.

Regarding quality metrics, the most striking finding was eSVD’s performance reversal from worst at moderate contamination (ARE = 0.23 at 0 dB) to best at extreme contamination (ARE = 0.99 at +20 dB). This reflects the trade-off between component retention strategy and signal-to-artifact ratio: conservative retention (SVD: 1–3 components) prioritizes specificity, whereas aggressive retention (eSVD: 4–5 components) ensures complete artifact capture when ECG dominates but risks incorporating neural features at moderate contamination. Given that moderate contamination (0–5 dB) is the most prevalent condition in subclavicular IPG placements [18] eSVD’s peak performance at extreme contamination addresses rare scenarios while failing in common conditions, rendering it unsuitable for robust aDBS deployment.

Beta power preservation emerged as the most clinically discriminative metric ($\eta_{p}^{2}$ = 0.644). Threshold-based aDBS systems [10,14] rely on accurate beta power measurement to determine stimulation adjustments. Systematic beta power underestimation, as exhibited by SVD at high contamination (BPP = 0.57 at +10 dB; 0.42 at +20 dB), would bias control algorithms toward under-stimulation, permitting breakthrough symptoms. While SVD showed slightly better performance at low contamination (BPP = 1.12 vs. 1.05 at 0 dB), TS demonstrated superior stability across increasing contamination levels (range 1.05–0.80) compared to SVD’s progressive degradation (range 1.12–0.42).

Regarding burst count preservation, the interpretation is more nuanced. At moderate contamination (0 dB), all methods showed comparable modest under-detection (BCP ≈ 0.94–0.98) without significant differences. At higher contamination levels, TS exhibited progressive over-detection (BCP = 1.26 at +10 dB; 1.69 at +20 dB), likely from residual QRS transients being misclassified as beta bursts, while other methods showed increasing under-detection. While TS exhibits inflation in burst detection at +20 dB, we caution that persistent over-detection would trigger unwarranted stimulation, raising the risk of stimulation-induced dyskinesias and accelerated battery drain. At such severe contamination levels (>15 dB), algorithmic cleaning alone may be insufficient, and clinical re-evaluation of IPG placement or recording contact selection should be prioritized. These findings suggest that burst-based aDBS control strategies may require adaptive thresholding or hybrid approaches to maintain accuracy under severe ECG contamination [32].

Furthermore, our evaluation utilized a fixed 75th percentile threshold to define pathological beta bursts, serving as a standardized offline comparative metric utilized by Stam et al. [19]. However, in clinical practice, fixed thresholds are vulnerable to the residual high-frequency noise that TS sometimes leaves behind at severe contamination levels, leading to burst over-detection. Future closed-loop implementations could greatly benefit from adaptive or dynamic thresholds—such as tracking the moving average of the local noise floor. By dynamically raising the burst detection threshold in response to localized residual ECG noise, control algorithms could effectively compensate for the imperfections of TS cleaning, minimizing false-positive pacing while maintaining therapeutic efficacy.

### 4.2. Clinical Implications

Our findings enable contamination-specific algorithm selection for individual patients. TS may be the recommended method for patients with moderate-to-high ECG contamination and subclavicular IPG placement, the predominant configuration in Percept implants [31]. TS’s superior real-time compatibility, stable beta power preservation across contamination levels (BPP range 1.05–0.80), and excellent timing consistency establish it as the default method for threshold-based aDBS applications [10,32].

For patients with minimal contamination, real-time SVD represents a validated alternative providing slightly superior beta power preservation at low contamination. However, this comes with the caveat of progressive degradation if contamination increases over time due to lead migration, tissue changes, or IPG repositioning. When recordings exhibit severe contamination, both TS and SVD show progressive quality degradation; alternative strategies should be prioritized: contact reconfiguration, temporary cranial IPG placement, or adoption of widened control thresholds accommodating estimation uncertainty. The Perceive toolbox is unsuitable for any aDBS application due to real-time incompatibility and severe beta power loss under high contamination [18,19].

TS’s demonstrated latencies enable seamless integration into existing aDBS control architectures. Little et al.’s [10] dual-threshold paradigm achieved 56% stimulation reduction using 400 ms windows with 100 ms updates. Our 7–11 ms processing times consume only 7–11% of the control cycle, leaving sufficient margin for downstream operations, including power computation, threshold comparison, and control decisions. For burst-based control strategies [6,23], while the processing latencies are within electrophysiological constraints (minimum burst duration 100–150 ms [6]), clinicians should be aware of the potential for over-detection at severe contamination levels and may need to implement compensatory measures such as burst rate consistency checks or hybrid power-burst control algorithms.

Beyond algorithm selection, robust aDBS implementation requires patient-specific optimization. Post-implantation evaluation should include: (i) Visual Inspection: timeline recordings should be inspected to estimate contamination severity and inform method selection [3,6]. (ii) Threshold Determination: individualized control thresholds should be based on cleaned beta power distributions during standardized motor tasks [10,32,33]. (iii) Long-Term Adaptation: adjustments may be necessary to accommodate signal changes from tissue encapsulation, impedance drift, and disease progression [18,31,34].

### 4.3. Limitations

Our study has some limitations. First, the simulated ground truth relied on the linear superposition of ECG and LFP signals. While mathematically necessary to quantify preservation metrics and methodologically aligned with recent standardizations by Stam et al. [19], this approach is a biophysical simplification that cannot perfectly replicate the complex volume conduction, spatial filtering, and dynamic tissue impedance effects of natural biological sources. However, by utilizing a medication-OFF baseline, our simulation framework provided a highly robust test of beta biomarker preservation compared to previous medication-ON simulations. To mitigate simulation limitations, we confirmed that the relative algorithmic performance rankings strongly converged (Spearman ρ = 0.92) when tested on our real-data clinical cohort, where true volume conduction effects were present. For real contaminated recordings, ground-truth clean signals were unavailable, necessitating reference-free convergence metrics rather than conventional quality assessment [27,28].

Second, our quantitative evaluation with a known ground truth relies on a limited sample size of only three patients (yielding 33 simulated test signals). While this highly restrictive selection criterion ensured the complete absence of natural ECG contamination, a mathematical prerequisite for accurate simulation, it inherently constrains the generalizability of our findings regarding baseline beta burst dynamics. Pathological burst rates and durations exhibit high inter-individual variability; consequently, a small sample size restricts the statistical power of comparisons involving highly variable temporal metrics, most notably the burst count preservation (BCP) metric. Due to this variance, small-to-moderate algorithmic distortions in burst counts may fail to reach statistical significance in a cohort of this size. Retrospective statistical power estimates suggest that detecting a moderate effect size (e.g., Cohen’s d = 0.5) in burst preservation differences between algorithms, with 80% power at an alpha level of 0.05, would require a minimum sample size of approximately 34 independent baseline recordings. Therefore, while our current simulation successfully establishes the fundamental signal processing trade-offs and frequency-domain preservation (BPP), the burst-domain findings (BCP) should be interpreted as preliminary. Future studies utilizing larger, multi-center datasets of artifact-free baseline recordings are required to adequately power these comparisons and definitively validate the generalizability of burst preservation across the broader Parkinson’s population.

Third, crucially, our computational benchmarking was performed on a standard PC workstation running MATLAB. We must explicitly state that the reported latencies represent PC-based comparative estimates rather than absolute embedded execution times. A standard PC architecture does not reflect the stringent physical realities and severe bottlenecks of a medical-grade embedded processor, which typically operates under a <10 mW power budget and is constrained by strict cache memory limits, real-time operating system (RTOS) overhead, and complex I/O interrupt handling. While TS’s linear O(N) computational complexity suggests it is mathematically suitable for low-power scaling, especially compared to the O(N^3^) complexity of SVD-based methods, the current results only establish theoretical comparative feasibility. Therefore, formal on-chip validation, for instance, deploying these algorithms on open-source investigational platforms for closed-loop neuromodulation (e.g., the OpenMind platform or equivalent translational hardware), is not merely a desirable extension but a strictly necessary future step before any clinical translation or device-level deployment can be considered [27,28].

Finally, our analysis was restricted to subcortical STN/GPi recordings in PD patients; validation in other disorders (essential tremor, dystonia, epilepsy) and recording sites is necessary before generalization, as different conditions may exhibit distinct baseline signal characteristics and biomarker requirements [32,35]. Finally, we employed offline 75th percentile thresholds for burst detection [6]; for real-time control where detection errors have asymmetric clinical consequences, optimal thresholds may require individualized adjustment [32,33].

## 5. Conclusions

This study identifies TS as the preferred solution for ECG artifact removal in real-time aDBS. TS proved capable of meeting the strict 100 ms control loop constraints, delivering P_99_ latencies of 7–11 ms while maintaining stable beta power preservation (BPP between 1.05 and 0.80) across clinically relevant contamination levels. Our findings challenge the assumption that more aggressive cleaning yields better biomarker estimation; instead, we demonstrate that balanced approaches prioritizing signal preservation achieve superior stability for neural control.

For clinical implementation, it is important to note that, while TS is highly robust, severe contamination may still risk burst over-detection. Consequently, future adaptive algorithms might benefit from hybrid control strategies that combine average beta power with burst metrics to maximize therapeutic fidelity. As sensing-enabled neurostimulators evolve into standard clinical tools, these findings establish essential engineering specifications for future hardware. By ensuring robust, real-time artifact removal, this work lays the groundwork for next-generation closed-loop systems capable of delivering precisely titrated, symptom-responsive therapy.

## Figures and Tables

**Table 1:** Computational performance across contamination levels.

Contamination	Method	Mean (ms)	P_99_ (ms)	Violations (%)	Status
**0 dB**	TS	5.58	9.56	0.29	Compatible
	SVD	6.19	13.27	3.04	Compatible
	eSVD	69.5	136.3	99.8	Incompatible
	PR	48.8	87.2	100	Incompatible
**+10 dB**	TS	5.69	9.49	0.79	Compatible
	SVD	5.87	13.37	1.04	Compatible
	eSVD	91.6	177.6	100	Incompatible
	PR	49.6	93.0	100	Incompatible
**+20 dB**	TS	6.25	10.73	2.64	Compatible
	SVD	5.29	9.15	0.96	Compatible
	eSVD	90.1	186.9	100	Incompatible
	PR	39.3	66.6	100	Incompatible

Note: Mean = average buffer processing time; P_99_ = 99th percentile worst-case latency (critical for real-time guarantees); Violations = percentage of buffers exceeding 50 ms threshold. Compatible status requires P_99_ ≤ 50 ms.

**Table 2:** Summary of method performance across contamination levels.

Method	0 dB			+10 dB			+20 dB		
	ARE	BPP	P_99_(ms)	ARE	BPP	P_99_(ms)	ARE	BPP	P_99_(ms)
**TS**	0.50	**1.05**	**9.56**	0.88	0.85	**9.49**	0.98	0.80	**10.73**
**SVD**	0.40	**1.12**	**13.27**	0.89	0.57	**13.37**	0.98	0.42	**9.15**
**eSVD**	0.23	1.31	**136.3**	0.92	**1.00**	**177.6**	0.99	0.75	**186.9**
**PR**	0.37	0.81	**87.2**	0.86	**0.29**	**93.0**	0.98	0.18	**66.6**

Notes: ARE = Artifact Removal Efficiency; BPP = Beta Power Preservation; P_99_ = 99th percentile latency. Real-time compatible methods (P_99_ ≤ 50 ms) shown in **bold**.

**Table 3:** Real data quality metrics (*n* = 36 recordings, medication states pooled).

Metric	TS	SVD	eSVD	PR	Significance
**ECG Suppression (dB)**	2.16 ± 1.44	3.22 ± 1.83	3.81 ± 3.50 *	1.09 ± 1.23	***
**Template Correlation**	0.36 ± 0.17	0.47 ± 0.16 **	0.49 ± 0.27 **	0.19 ± 0.13	***
**Beta Peak Recovery**	0.95 ± 0.57	0.91 ± 0.64	0.85 ± 0.58	0.86 ± 0.57	n.s.
**Power Law Score**	0.47 ± 0.14	0.49 ± 0.13	0.56 ± 0.19	0.52 ± 0.15	n.s.

Note: Values = mean ± SD. * = *p* < 0.05 vs. TS; ** = *p* < 0.01 vs. non-SVD methods. Significance: *** = *p* < 0.001 with significant pairwise differences; n.s. = not significant.

**Table 4:** Real data computational performance (*n* = 18 patients, 36 bilateral recordings).

Method	Channel	M0 Mean (ms)	OFF P_99_ (ms)	M1 Mean (ms)	ON P_99_ (ms)	Change	Violations
TS	Left	4.13 ± 0.46	8.58	3.69 ± 0.27	7.13	−17%	<0.5%
TS	Right	3.99 ± 0.29	7.83	3.87 ± 0.28	7.45	−5%	<0.5%
SVD	Left	4.74 ± 1.18	9.17	3.83 ± 0.36	7.22	−21%	<0.8%
SVD	Right	4.07 ± 0.29	8.42	3.80 ± 0.09	6.96	−17%	<0.8%

Note: Mean = average buffer processing time; P_99_ = 99th percentile worst-case latency (critical for real-time guarantees); Change = percentage reduction in P_99_ from M0 to M1 state; Violations = percentage of buffers exceeding 50 ms threshold. Inter-hemispheric P_99_ differences <1 ms.

## Data Availability

The data presented in this study are available on request from the corresponding author due to privacy and ethical restrictions regarding patient confidentiality.

## Abbreviations

The following abbreviations are used in this manuscript:

aDBS	Adaptive Deep Brain Stimulation
ARE	Artifact Removal Efficiency
BCP	Burst Count Preservation
BPP	Beta Power Preservation
cDBS	Continuous Deep Brain Stimulation
DBS	Deep Brain Stimulation
ECG	Electrocardiogram
FFT	Fast Fourier Transform
GPi	Globus Pallidus Internus
IPG	Implanted Pulse Generator
LFP	Local Field Potential
PD	Parkinson’s Disease
PR	Perceive Toolbox Method
PSD	Power Spectral Density
RMS	Root Mean Square
SPR	Signal Preservation Ratio
STN	Subthalamic Nucleus
SVD	Singular Value Decomposition
eSVD	Extended Singular Value Decomposition
TS	Template Subtraction

## References

[1] Deuschl G., Schade-Brittinger C., Krack P., Volkmann J., Schäfer H., Bötzel K., Daniels C., Deutschländer A., Dillmann U., Eisner W. (2006). A Randomized Trial of Deep-Brain Stimulation for Parkinson’s Disease A BS TR AC T. N. Engl. J. Med..

[2] Weaver F.M., Follett K., Stern M., Hur K., Harris C., Marks W.J., Rothlind J., Sagher O., Reda D., Moy C.S. (2009). Bilateral Deep Brain Stimulation vs Best Medical Therapy for Patients with Advanced Parkinson Disease: A Randomized Controlled Trial. JAMA.

[3] Kühn A.A., Kupsch A., Schneider G.H., Brown P. (2006). Reduction in Subthalamic 8–35 Hz Oscillatory Activity Correlates with Clinical Improvement in Parkinson’s Disease. Eur. J. Neurosci..

[4] Kühn A.A., Kempf F., Brücke C., Doyle L.G., Martinez-Torres I., Pogosyan A., Trottenberg T., Kupsch A., Schneider G.H., Hariz M.I. (2008). High-Frequency Stimulation of the Subthalamic Nucleus Suppresses Oscillatory β Activity in Patients with Parkinson’s Disease in Parallel with Improvement in Motor Performance. J. Neurosci..

[5] Thenaisie Y., Palmisano C., Canessa A., Keulen B.J., Capetian P., Jiménez M.C., Bally J.F., Manferlotti E., Beccaria L., Zutt R. (2021). Towards Adaptive Deep Brain Stimulation: Clinical and Technical Notes on a Novel Commercial Device for Chronic Brain Sensing. J. Neural Eng..

[6] Tinkhauser G., Pogosyan A., Tan H., Herz D.M., Kühn A.A., Brown P. (2017). Beta Burst Dynamics in Parkinson’s Disease off and on Dopaminergic Medication. Brain.

[7] Tinkhauser G., Moraud E.M. (2021). Controlling Clinical States Governed by Different Temporal Dynamics with Closed-Loop Deep Brain Stimulation: A Principled Framework. Front. Neurosci..

[8] Tinkhauser G., Torrecillos F., Duclos Y., Tan H., Pogosyan A., Fischer P., Carron R., Welter M.-L., Karachi C., Vandenberghe W. (2018). Beta Burst Coupling across the Motor Circuit in Parkinson’s Disease. Neurobiol. Dis..

[9] Priori A., Foffani G., Rossi L., Marceglia S. (2013). Adaptive Deep Brain Stimulation (ADBS) Controlled by Local Field Potential Oscillations. Exp. Neurol..

[10] Little S., Pogosyan A., Neal S., Zavala B., Zrinzo L., Hariz M., Foltynie T., Limousin P., Ashkan K., FitzGerald J. (2013). Adaptive Deep Brain Stimulation in Advanced Parkinson Disease. Ann. Neurol..

[11] van Rheede J.J., Feldmann L.K., Busch J.L., Fleming J.E., Mathiopoulou V., Denison T., Sharott A., Kühn A.A. (2022). Diurnal Modulation of Subthalamic Beta Oscillatory Power in Parkinson’s Disease Patients during Deep Brain Stimulation. npj Park. Dis..

[12] Arlotti M., Marceglia S., Foffani G., Volkmann J., Lozano A.M., Moro E., Cogiamanian F., Prenassi M., Bocci T., Cortese F. (2018). Eight-Hours Adaptive Deep Brain Stimulation in Patients with Parkinson Disease. Neurology.

[13] Sanger Z.T., Henry T.R., Park M.C., Darrow D., McGovern R.A., Netoff T.I. (2024). Neural Signal Data Collection and Analysis of Percept^TM^ PC BrainSense Recordings for Thalamic Stimulation in Epilepsy. J. Neural Eng..

[14] Stanslaski S., Summers R.L.S., Tonder L., Tan Y., Case M., Raike R.S., Morelli N., Herrington T.M., Beudel M., Ostrem J.L. (2024). Sensing Data and Methodology from the Adaptive DBS Algorithm for Personalized Therapy in Parkinson’s Disease (ADAPT-PD) Clinical Trial. npj Park. Dis..

[15] Bronte-Stewart H.M., Beudel M., Ostrem J.L., Little S., Almeida L., Ramirez-Zamora A., Fasano A., Hassell T., Mitchell K.T., Moro E. (2025). Long-Term Personalized Adaptive Deep Brain Stimulation in Parkinson Disease. JAMA Neurol..

[16] Wilkins K.B., Melbourne J.A., Akella P., Bronte-Stewart H.M. (2023). Unraveling the Complexities of Programming Neural Adaptive Deep Brain Stimulation in Parkinson’s Disease. Front. Hum. Neurosci..

[17] Busch J.L., Kaplan J., Behnke J.K., Witzig V.S., Drescher L., Habets J.G.V., Kühn A.A. (2025). Chronic Adaptive Deep Brain Stimulation for Parkinson’s Disease: Clinical Outcomes and Programming Strategies. npj Park. Dis..

[18] Neumann W.J., Memarian Sorkhabi M., Benjaber M., Feldmann L.K., Saryyeva A., Krauss J.K., Contarino M.F., Sieger T., Jech R., Tinkhauser G. (2021). The Sensitivity of ECG Contamination to Surgical Implantation Site in Brain Computer Interfaces. Brain Stimul..

[19] Stam M.J., van Wijk B.C.M., Sharma P., Beudel M., Piña-Fuentes D.A., de Bie R.M.A., Schuurman P.R., Neumann W.-J., Buijink A.W.G. (2022). A Comparison of Methods to Suppress Electrocardiographic Artifacts in Local Field Potential Recordings. Clin. Neurophysiol..

[20] Hammer L.H., Kochanski R.B., Starr P.A., Little S. (2022). Artifact Characterization and a Multipurpose Template-Based Offline Removal Solution for a Sensing-Enabled Deep Brain Stimulation Device. Ster. Funct. Neurosurg..

[21] Swinnen B.E.K.S., Buijink A.W.G., Stam M.J., Hubers D., de Neeling M., Keulen B.J., Morgante F., van Wijk B.C.M., de Bie R.M.A., Ricciardi L. (2025). Pitfalls and Practical Suggestions for Using Local Field Potential Recordings in DBS Clinical Practice and Research. J. Neural Eng..

[22] Chen Y., Ma B., Hao H., Li L. (2021). Removal of Electrocardiogram Artifacts From Local Field Potentials Recorded by Sensing-Enabled Neurostimulator. Front. Neurosci..

[23] Tinkhauser G., Pogosyan A., Little S., Beudel M., Herz D.M., Tan H., Brown P. (2017). The Modulatory Effect of Adaptive Deep Brain Stimulation on Beta Bursts in Parkinson’s Disease. Brain.

[24] Canessa A., Palmisano C., Isaias I.U., Mazzoni A. (2020). Gait-Related Frequency Modulation of Beta Oscillatory Activity in the Subthalamic Nucleus of Parkinsonian Patients. Brain Stimul..

[25] Welch P. (1967). The Use of Fast Fourier Transform for the Estimation of Power Spectra: A Method Based on Time Averaging over Short, Modified Periodograms. IEEE Trans. Audio Electroacoust..

[26] Donoghue T., Haller M., Peterson E.J., Varma P., Sebastian P., Gao R., Noto T., Lara A.H., Wallis J.D., Knight R.T. (2020). Parameterizing Neural Power Spectra into Periodic and Aperiodic Components. Nat. Neurosci..

[27] Afshar P., Khambhati A., Stanslaski S., Carlson D., Jensen R., Linde D., Dani S., Lazarewicz M., Cong P., Giftakis J. (2013). A Translational Platform for Prototyping Closed-Loop Neuromodulation Systems. Front. Neural Circuits.

[28] Rodriguez-Zurrunero R., Araujo A., Lowery M.M. (2021). Methods for Lowering the Power Consumption of OS-Based Adaptive Deep Brain Stimulation Controllers. Sensors.

[29] Cummins D.D., Kochanski R.B., Gilron R., Swann N.C., Little S., Hammer L.H., Starr P.A. (2021). Chronic Sensing of Subthalamic Local Field Potentials: Comparison of First and Second Generation Implantable Bidirectional Systems Within a Single Subject. Front. Neurosci..

[30] Gilron R., Little S., Perrone R., Wilt R., de Hemptinne C., Yaroshinsky M.S., Racine C.A., Wang S.S., Ostrem J.L., Larson P.S. (2021). Long-Term Wireless Streaming of Neural Recordings for Circuit Discovery and Adaptive Stimulation in Individuals with Parkinson’s Disease. Nat. Biotechnol..

[31] Neumann W.-J., Staub-Bartelt F., Horn A., Schanda J., Schneider G.-H., Brown P., Kühn A.A. (2017). Long Term Correlation of Subthalamic Beta Band Activity with Motor Impairment in Patients with Parkinson’s Disease. Clin. Neurophysiol..

[32] Little S., Brown P. (2020). Debugging Adaptive Deep Brain Stimulation for Parkinson’s Disease. Mov. Disord..

[33] Neumann W.-J., Turner R.S., Blankertz B., Mitchell T., Kühn A.A., Richardson R.M. (2019). Toward Electrophysiology-Based Intelligent Adaptive Deep Brain Stimulation for Movement Disorders. Neurotherapeutics.

[34] Feldmann L.K., Neumann W.J., Krause P., Lofredi R., Schneider G.H., Kühn A.A. (2021). Subthalamic Beta Band Suppression Reflects Effective Neuromodulation in Chronic Recordings. Eur. J. Neurol..

[35] Little S., Brown P. (2012). What Brain Signals Are Suitable for Feedback Controzl of Deep Brain Stimulation in Parkinson’s Disease?. Ann. N. Y. Acad. Sci..

